# Dermatopathology of Orf Virus (Malaysian Isolates) in Mice Experimentally Inoculated at Different Sites with and without Dexamethasone Administration

**DOI:** 10.1155/2018/9207576

**Published:** 2018-08-01

**Authors:** Jamilu Abubakar Bala, Krishnan Nair Balakrishnan, Ashwaq Ahmed Abdullah, Tay Kimmy, Yusuf Abba, Ramlan Bin Mohamed, Faez Firdaus Abdullah Jesse, Abd Wahid Haron, Mustapha Mohamed Noordin, Asinamai Athliamai Bitrus, Idris Umar Hambali, Mohd Azmi Mohd Lila

**Affiliations:** ^1^Department of Pathology and Microbiology, Faculty of Veterinary Medicine, Universiti Putra Malaysia, 43400 Serdang, Selangor, Malaysia; ^2^Microbiology Unit, Department of Medical Laboratory Science, Faculty of Allied Health Sciences, Bayero University Kano, P.M.B. 3011, Kano, Nigeria; ^3^Institute of Bioscience, University Putra Malaysia, 43400 Serdang, Selangor DarulEhsan, Malaysia; ^4^Department of Microbiology, Faculty of Applied Science, Taiz University, Taiz, Yemen; ^5^Department of Veterinary Clinical Studies, Faculty of Veterinary Medicine, Universiti Putra Malaysia, 43400 UPM Serdang, Selangor, Malaysia; ^6^Department of Veterinary Pathology, Faculty of Veterinary Medicine, University of Maiduguri, PMB 1069, 600233, Borno State, Nigeria; ^7^Institut Penyelidikan Haiwan, (IPH), Veterinary Research Institute, Ipoh, 59, Jalan Sultan Azlan Shah, 31400 Ipoh, Perak, Malaysia; ^8^Research Centre for Ruminant Diseases, Faculty of Veterinary Medicine, Universiti Putra Malaysia, 43400 UPM Serdang, Selangor, Malaysia

## Abstract

Orf is a clinical manifestation of parapoxvirus infection often fatal in goats and sheep especially when they are under stress or influenced by unfavorable environment. This study investigated the pathogenicity of two Orf virus isolates (ORFV UPM1/14 and UPM2/14) and host response in mouse model by using different inoculation sites with/without prior exposure to dexamethasone. Treatments with dexamethasone served as an immunosuppressant that may mimic stress situation in affected animals. Groups of five mice were given intradermal injection of 0.2 mL of tissue culture infective dose 50 (TCID_50_) of UPM1/14 (Group 1) and UPM2/14 (Group 2) at the dorsum (Group 1A; Group 2A), ear pinna (Group 1B; Group 2B), and labial commissure (Group 1C; Group 2C). An inoculum 0.2 mL of UPM1/14 was administered to animals treated with dexamethasone (n=5; 5 mg/kg/day intraperitoneally) and nondexamethasone (n=5) groups at the dorsum, ear pinna, and labial commissure. No significant difference (p>0.05) was observed in the mean lesion scores among the groups of different inoculation sites or between dexamethasone-treated and nontreated groups. However, there was a significant difference (p<0.05) in the mean stratum thickness of affected skin following inoculation with UPM2/14 isolate at the ear pinna and labial commissure. Histopathology examination revealed keratosis, acanthosis, and ballooning degeneration in the skin of affected mice. Orf virus DNA was detected in the skin samples by targeting F1L and B2L virus-specific genes in polymerase chain reaction (PCR) assay. Intradermal inoculation with UPM1/14 or UPM2/14 isolate produced a mild skin lesion in mice, and there was no significant difference in orf disease manifestation despite variation of inoculation sites. Similarly, short-term dexamethasone administration gave no adverse effects on pathogenicity of orf virus isolates.

## 1. Introduction

Orf virus (ORFV) belongs to the genus* Parapoxvirus* (PPV) of the family Poxviridae [1]. It is the etiological agent of contagious ecthyma (CE), a severe exanthematic dermatitis that affects domestic and wild small ruminants [2]. Orf has also been reported in camels and camelids, members of the Cervidae family, and various other ruminants. Dogs, cats, and squirrels can also become infected with the orf virus [3, 4]. Based on a recent seroprevalence study, evaluation of IgM antibodies in small ruminant populations showed higher incidence in goats than sheep [5]. The disease has a zoonotic potential particularly for those who are in close contact with animals such as veterinarians, farmers, animal attendants, and visitors [6–8].

Orf virus usually gains access to the host's tissue through injury, breaks, and abrasions in the skin and replicates in regenerating epidermal keratinocytes [9]. The viral replication resulted in edematous and granulomatous inflammation of the dermal cells [3]. Orf is clinically recognized by the appearance of papules, vesicles, pustules, and rapidly growing scabs which are confined to the lips and muzzle of the affected animals [10]. Contagious ecthyma is not usually lethal, and lesions typically resolve within 2 to 4 weeks; however, death may result if secondary complications such as bacterial infections or myiasis develop [11].

Morbidity rates from CE can reach up to 70% in flocks where the disease is occurring for the first time [6, 12]. Besides disruption of the national and international trade of animal and animal products, the lesions of CE can also affect the optimum productivity of animals and reduce the market value of meat, leather and, wool [8, 13].

In immunocompromised animals, extensive and recurrent Orf lesions may occur. This will undoubtedly have resulted in economic losses to small livestock farmers. Although gross clinical signs can be used as a good reference for the diagnosis of this disease, however, the gold standard is to carry out virus isolation and molecular detection similarly for many other viruses ([14] Balakrishnan et al., 2017).

According to Cargnelutti et al. [10], clinical lesions were successfully reproduced accompanied by virus isolation in mice inoculated with ORFV. Recently, isolation of caprine ORFV in Malaysia provided further information about its relationship with other ORFV isolates from other parts of the world [6]. Meanwhile a recent study in rat infection model showed that differences in inoculation sites and induction of immune suppression using dexamethasone were observed to demonstrate varying pathological responses in rats [15]. Apart from the cytopathic effect of the virus in vitro, there is dearth of information on its pathogenicity in mouse model.

Dexamethasone inhibited the expression of proinflammatory mediators produced by antigen-presenting cells and is widely used clinically for treating a range of inflammatory disorders, e.g., allergies, asthma, autoimmune diseases, and sepsis [16, 17]. The effects of dexamethasone especially glucocorticoids are pleiotropic and mediated through mechanisms involving direct gene activation via binding to glucocorticoid-responsive genomic elements and indirect effects through interactions with transcription factors and by the modulation of signaling molecule; thus it is a well-known phenomenon that immunosuppressive agents aggravate disease repercussion in both natural and experimental conditions [18, 19].

In this study, we examined the development of orf disease lesion and its severity following infection with ORFV isolates in naïve host and upon treatment with dexamethasone. The pathogenicity of two Malaysian ORFV isolates in a mouse model was evaluated by using different inoculation sites and with/without administration of dexamethasone. This study provides valuable information on the pathogenicity of Malaysian strains of ORFV for future studies in its natural host.

## 2. Materials and Methods

### 2.1. Sample Processing and Virus Titration (TCID_50_)

Scab samples from infected goats were homogenized in 10% Phosphate Buffered Saline (PBS). The suspension was then centrifuged at 250xg 1000 rpm for 10 minutes and the supernatant was collected. Antibiotics were added to the supernatant at a concentration of 10,000 units/mL Penicillin and 10,000 *μ*g/mL Streptomycin (HyClone^®^) [6] were stored at −80°C for further use. The virus in the supernatant was subjected to titration by means of tissue culture infective dose 50% (TCID50) where serial ten dilutions (10^−1^ to 10^−10^) were made and 100uL of each dilution was added onto the confluent LT (lamb testis) monolayer cells in a 24-well culture plate. The plate was incubated with the virus suspension at 37°C for 1 hour and washed with sterile PBS before addition of fresh DMEM media containing 1% FBS. The plate was incubated at 37°C and observed daily for evidence of cytopathic effect (CPE) in the cells. After 1 week of incubation, wells showing visual evidence of CPE in all dilutions were recorded and TCID_50_ was estimated using a method previously described by Reed and Muench (1938).

(1) $$\begin{array}{c} Proportionate\;\;Distance=\frac{\left(\%\;\;CPE\;\;at\;\;dilution\;\;next\;\;above\;\;50\%\right)-50\%}{\left(\%\;\;CPE\;\;at\;\;next\;\;dilution\;\;above\;\;50\%\right)-\left(\%\;\;CPE\;\;at\;\;next\;\;dilution\;\;below\;\;50\%\right)} \end{array}$$

### 2.2. Animal Management

Four-week-old Balb/c mice weighing 9 grams to 11 grams were used in this study. Before the commencement of all procedures, mice were confirmed to be free from Orf virus and tested to be negative for anti-orf virus antibodies using enzyme-linked immunosorbent assay (ELISA). Mice were fed ad libitum.

### 2.3. Ethical Approval

All experimental procedures were performed according to the Guidelines for the Care and Use of Laboratory Animals approved by Institutional Animal Care and use Committee IACUC, Universiti Putra Malaysia (Ref: UPM/IACUC/FYP.2015/FPV.052). Humane endpoints were chosen to minimize or terminate pain or distress in the animals via euthanasia.

### 2.4. Experimental Design

#### 2.4.1. Experiment 1

**Figure 1:**
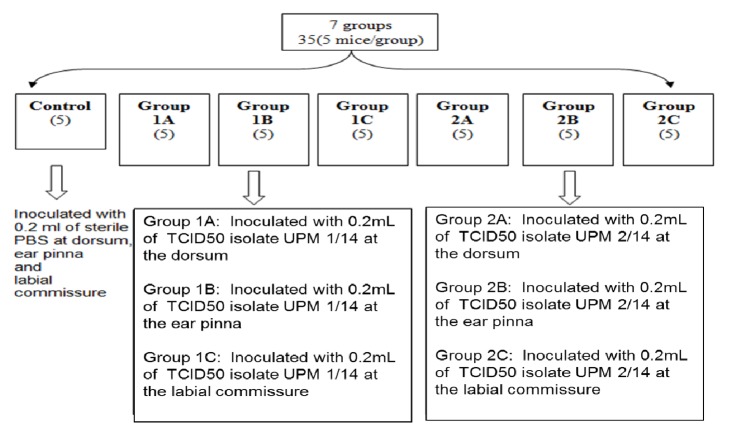
The design of Experiment 1.

A total of thirty-five (35) mice were used. Five mice used as control group were inoculated intradermally with 0.2 mL of sterile Phosphate Buffered Saline (PBS) at the dorsum, ear pinna, and labial commissure. These are among the common sites of lesion occurrence in CE to mimic orf disease in its natural host. The remaining 30 mice were divided into two equal groups of 15 mice each, Group 1 (UPM1/14 inoculation) and Group 2 (UPM2/14 inoculation). Five mice in each group were injected intradermally with 0.2 mL of TCID_50_ of UPM1/14 and UPM2/14 at the dorsum (Group 1A; Group 2A), ear pinna (Group 1B; Group 2B), and labial commissure (Group 1C; Group 2C). The mice were monitored for 14 days and euthanized later by using Ketamine/Xylazine at 50 mg/kg + 5 mg/kg.  Figure 1 summarized the flowchart of the animal inoculation and groupings.

#### 2.4.2. Experiment 2

**Figure 2:**
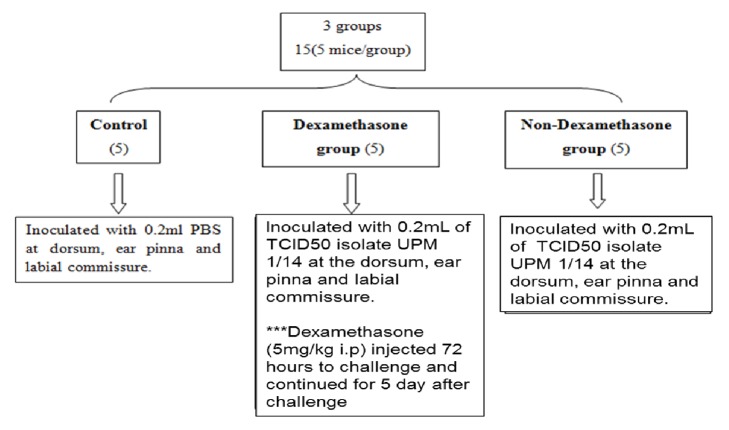
The design of Experiment 2.

The effect of dexamethasone administration on the pathogenicity of UPM1/14 ORFV isolate was evaluated. Fifteen (15) mice were used for this study; 5 mice serving as control group were inoculated with 0.2 mL sterile PBS intradermally at the dorsum, ear pinna, and labial commissure. The remaining 10 mice were divided into two groups of 5 mice each; Dexamethasone group and non-Dexamethasone group. Mice in both groups were inoculated with 0.2 mL of TCID_50_ of UPM1/14 isolate intradermally at the dorsum, ear pinna, and labial commissure. Mice in the dexamethasone group were injected with dexamethasone (5 mg/kg/day) intraperitoneally 3 days prior to viral inoculation and continued for 5 days after viral challenge (Figure 2). All mice were observed for 6 days and euthanized by using Ketamine/xylazine at 50 mg/kg + 5 mg/kg dosage.

### 2.5. Clinical Scoring

All the animals were monitored on daily basis for the development of lesions characteristics of orf virus disease (hyperaemia, vesicles, pustules, and scabs) and clinical signs including ruffled hair coat, responsiveness and ocular discharges were assessed. Each indicator was scored as 0 (absence), 1 (mild), 2 (moderate), and 3 (severe); Table 1 showed the detailed description of scoring protocol employed for assessment of the relevant clinical signs.

### 2.6. Histopathological Examination

Skin tissues from infected mice were collected in 10% buffered formalin, processed, embedded, and sectioned. Hematoxylin and Eosin (H&E) procedure for demonstrating general morphology of the skin was carried out for all the processed samples. The slides were viewed at ×100, 200 and 400 magnifications and images were captured by using Moticam Pro 285A as previously reported [5, 15]. Morphological changes in linings epithelial cells of the skin were observed and recorded accordingly. Thickness of the stratum basale layer was measured and mean was calculated for all the samples from both UPM 1/14 and 2/14 and results were tabulated.

### 2.7. DNA Extraction and Polymerase Chain Reaction

Skin tissues from infected mice were collected for DNA extraction by using Vivantis GF-1, a commercial standard DNA extraction kit. Virus suspensions were prepared from each sample and DNA was extracted by using protocol previously described by Abdullah et al. [6, 20]. DNA extracted was stored at −20°C until further use.

Standard PCR was carried out targeting the most immunogenic proteins of the virus, namely, B2L gene and F1L gene (Abdullah et al., 2015a; [14, 21]). These genes coding structural and valuable for the replication process of the ORFV. The PCR procedure was previously described [20] by using primer sets: B2L and F1L (B2L forward primer:5-ATG TGG CCG TTC TCC TCT ATC-3; B2L reverse primer:5-TTA ATT TAT TGG CTT GCA G-3; F1L forward primer: 5-ATG GAT CCA CCC GAA ATC AC-3; and F1L reverse primer: 5-TCA CAC GAT GGC CGT GAC CAG-3). PCR procedure was accomplished according to the manufacturer's instruction (Novagen, Toyobo, Japan). Thermal cycler was programmed according to the following conditions: 95°C for 2 min as an initial activation step, followed by 35 cycles of 94°C for 20s; 50°C for 30s, 70°C for 20s, and one final cycle of 72°C for 2 min. PCR amplicons were run in the agarose gel and electrophoresed at 100 V for 50 min. The gel was stained in* Red Safe* Nucleic Acid Staining Solution and DNA bands were viewed in gel documentation system.

### 2.8. Statistical Analysis

Data collected for lesion scores, clinical signs scores, and stratum thickness were summarized as mean ±S.E and subjected to statistical analysis using IBM SPSS Statistics 20. One-way ANOVA/Kruskal-Wallis H test, at P<0.05, was performed.

## 3. Results

### 3.1. Virus Growth and Titres

**Figure 3:**
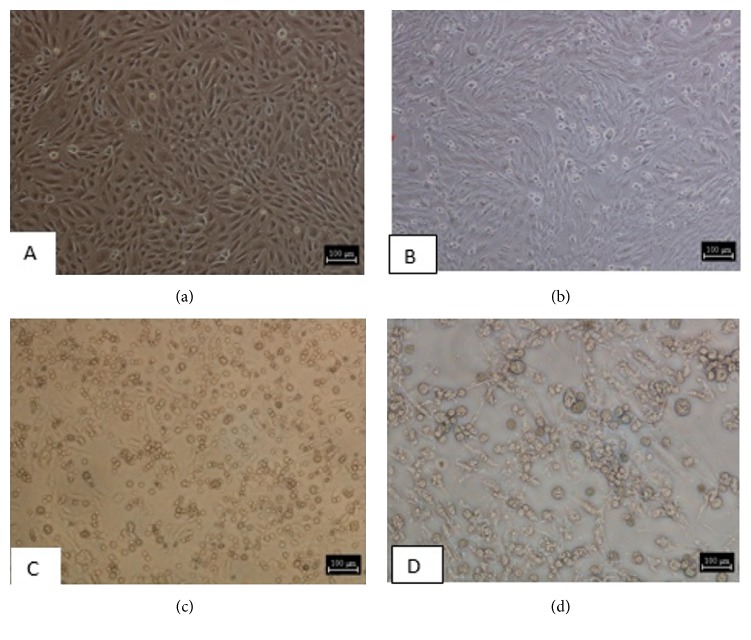
**Cytopathic effects following ORFV in LT cells**. (a) is uninoculated well of the LT cells. Plates (b), (c), and (d) showed infected cells with cytopathic effects after 24, 72, and 144 hours after inoculation, respectively.

ORFV was grown in LT cells and it was monitored daily for development of cytopathic effects. Virus titres determined by (TCID_50_) were 10^8.1^/ml for UPM 1/14 and 10^7.2^/ml for UPM 2/14. Various stages of the CPE development following virus inoculation were shown as in Figure 3.

### 3.2. Clinical Signs

**Figure 4:**
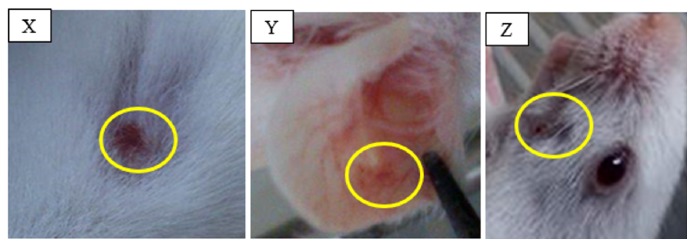
Development of Orf lesions at inoculations sites where mild hyperemic lesions of the infected mice, X, Y, and Z were respective dorsum, ear pinna, and labial commissure.

Scabs formation was observed at the inoculation sites of mice inoculated at the dorsum and labial commissure after 5 days of inoculation. None of the mice exhibited clinical signs such as reduced responsiveness, ruffled hair coat, and presence of ocular discharges.  Figure 4 shows a representative typical lesion of infected mice at the three inoculation sites.

### 3.3. Mean Lesion Score

**Figure 5:**
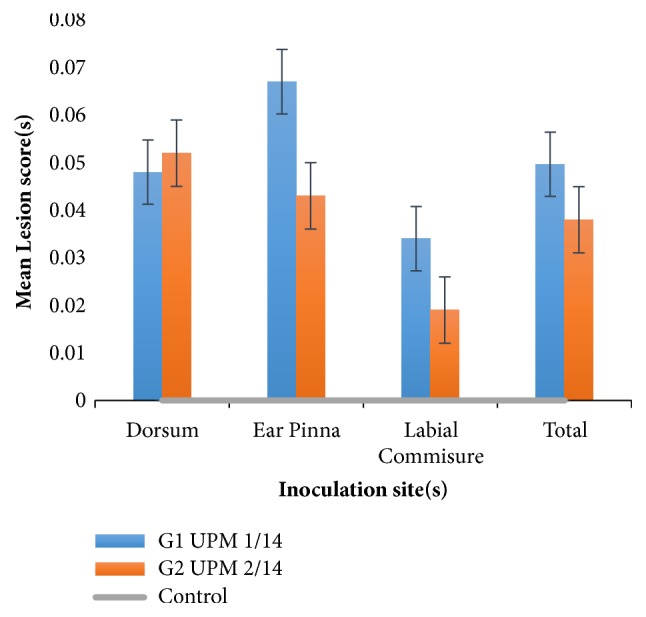
Distribution of mean lesion scoring and standard error in groups 1 and 2.

The highest mean lesion measurement of 0.067 ± 0.020 was observed for ear pinna of UPM 1/14 followed by dorsal mean lesion of 0.052 ± 0.017 for UPM 2/14 and the least measurement of 0.019 ± 0.008 was observed at the labial commissure of UPM 2/14. In general, the mean lesion scores observed from all the inoculation sites of UPM 1/14 and UPM 2/14 groups were not significantly different (p>0.05) (Table 2). Similarly, the total mean lesion scores between the two inoculation groups were not significantly different (p>0.05) (Table 3). Based on this observation, the two isolates may have similar pathogenicity.  Figure 5 shows the distribution of mean lesion scoring of each group of mice.

The mean lesion measurement for ruffled hair coat and alertness was found to be of 0.311 ± 0.108 and 0.292 ± 0.136, respectively, for dexamethasone-treated group, while zero measurements were recorded for both indicators in the non-dexamethasone-treated groups (Table 4); however, the mean score for clinical signs was significantly higher in the dexamethasone-treated group than in the nontreated group but this difference was not statistically significant (p<0.05).

However, there was no significant difference (p>0.05) in the total mean lesion scores between the dexamethasone-treated and -nontreated groups (Table 5).

### 3.4. Histopathology Lesion

**Figure 6:**
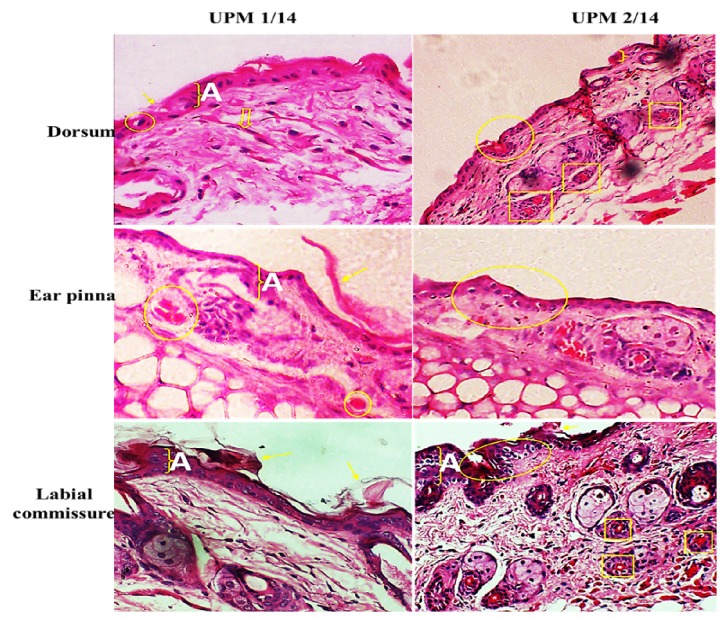
Histopathology section of the skin (dorsum, ear pinna, and labial commissure) of mice inoculated with ORFV UPM1/14 and UPM2/14, showing acanthosis (A), ballooning degeneration and vascular congestion (yellow circle), keratosis (yellow arrows), Langerhans cells (big down arrow), and vasculitis (yellow square), H&E × 200.

In general, keratosis, acanthosis, and ballooning degeneration were frequently observed in all experimental groups. Vascular congestion and vasculitis were observed in the ear pinna and labial commissure, respectively. Inflammatory cells typified by lymphocytes and neutrophils and Langerhans cells were also observed in some skin sections (Figure 6).

The highest mean stratum thickness of 34.30 ± 1.44 was observed in the labial commissure of the group 2 experiment; this is followed by dorsum 27.20 ± 2.85 of the same group 2 experiment; the least mean stratum thickness 13.0 ± 0.17 was found in the ear pinna of group 1 experiment. In general, it showed that the mean stratum thickness was higher (p<0.05) in the skin of the ear and labial commissure of UPM 2/14 inoculation group and comparable (p>0.05) in the dorsum of both inoculation groups (Table 6). There was no statistical significant difference despite this variation of the observed measurements.

**Figure 7:**
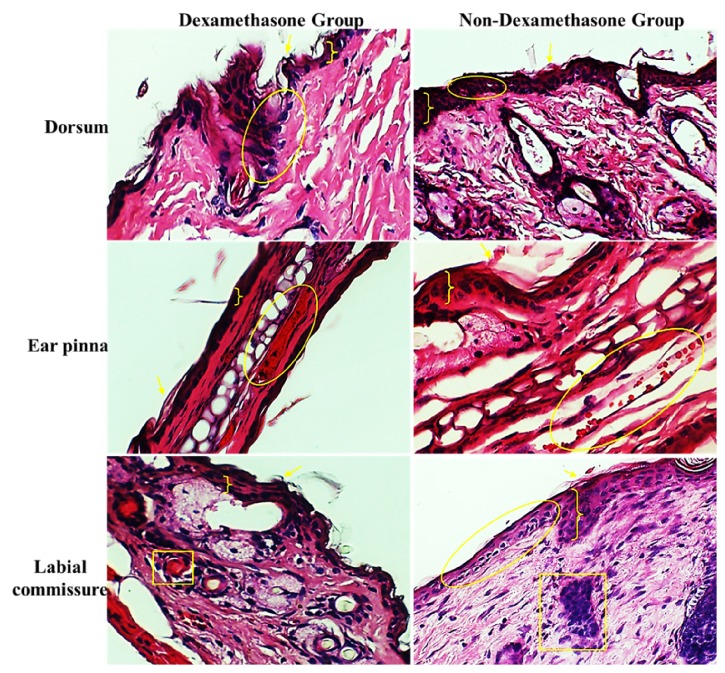
Histopathology section of the skin (dorsum, ear pinna, and labial commissure) of mice inoculated with UPM 1/14 with and without dexamethasone treatment, showing acanthosis ( }), ballooning degeneration and vascular congestion (yellow circle), and vasculitis (yellow square), H&E × 200.

Histopathology sections of infected tissues from mice treated with/without dexamethasone are presented in Figure 7. As reported earlier, there was no difference in lesion scores between the two groups. Thus, the histopathology lesions observed in these mice were similar to that of the groups inoculated with ORFV UPM1/14 and lesions observed included acanthosis, ballooning degeneration, and vasculitis.

### 3.5. Detection of ORFV Specific Genes by PCR Amplification

**Figure 8:**
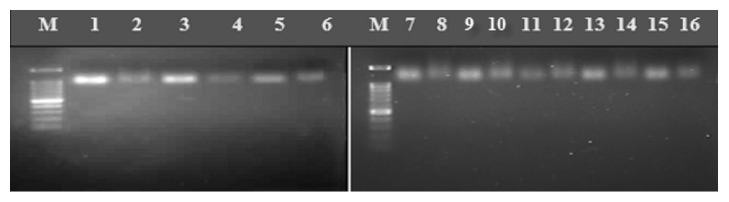
Lane M: 100 bp DNA ladder RTU (GeneDireX) DNA marker; Lanes 1 and 2: positive control; Lanes 3 and 4: sample from dorsum (Group 1); Lanes 5 and 6: sample from dorsum (Group 2); Lanes 7, and 8: positive control; Lane 9 and 10: sample from ear pinna (Group 1); Lanes 11 and 12: sample from ear pinna (Group 2); Lanes 13 and 14: sample from labial commissure (Group 1); Lanes 15 and 16: sample from labial commissure (Group 2). (Lanes 1, 3, 5, 7, 9, 11, 13, and 15: F1L primer; Lanes 2, 4, 6, 8, 10, 12, 14, and 16: B2L primer).

Skin tissue samples from all groups following different sites of inoculation with ORFV isolates showed positive PCR amplification DNA bands at 1062 bp (representing F1L gene) and 1199 bp (representing B2L gene) in size (Figure 8).

## 4. Discussion

Generally, mice inoculated with ORFV UPM 1/14 or UPM 2/14 isolates demonstrated development and formation of orf disease related scab at dorsum and labial commissure of mice beginning from 5-day postvirus inoculation. Interestingly, histopathology examination showed that the type of lesions developed in mice was not exactly akin to the lesions that were normally observed in its natural host vis-a-vis goats and sheep. The lesions developed from the establishment of erythema and progress to formation of macule, papules, vesicles, pustules, and scab. The similar lesion progression had been observed and described in other studies by Cargnelutti et al., 2011 [10], and Abbas & Mughal, 2014 [22], reported the formation of erythema, macule, and scab, with seldom observations of papules and vesicles in mice. Huda et al. [23] observed that blisters and crust were fully developed 4 days after virus inoculation and lasted for a longer period than what was observed in this study. It is believed that these variations occurred due to the difference in the strain of the virus for infection studies or because of immune variations in the strain of laboratory mouse used. The occurrence and recapitulation of orf and many other viral diseases especially in experimental set-up can be influenced by multiple factors such as age, breed, and strain of animal used as a virus infection model [4, 24].

The severity of skin lesions observed was similar across the various inoculation sites. Histopathology showed no difference in the mean lesion scores among the three inoculation sites. In goats and sheep, Orf lesions were often observed at the labial commissure, which is usually exposed to spiny forages that might produce abrasions during grazing, allowing the virus to penetrate the skin and replicate [6, 13]. Although there were no differences in the total mean lesion scores, there was a significant difference in the mean stratum thickness which induced the two UPM ORFV isolates following inoculation on the ear pinna and labial commissure. Dorsum, ear pinna, and labial commissure are the common sites where lesions manifest in goats and other small ruminants. Therefore, dermatopathology assessment by using these inoculation sites is important and appropriate for determination of virus pathogenicity and accurate measurement of host cellular response in animal infection model. As per other studies, this approach is practical and very common for measurement immediate host response to many viruses and microbial agents [25, 26]. In the previous study using rat model, it was observed that a higher mean lesion score was in the skin of rats inoculated with ORFV UPM 1/14 [15]. The difference was not observed in mouse model and the phenomenon could be attributed to the difference in animal species. Since ORFV is an epitheliotropic virus which replicates in new proliferative keratinocyte population, upon entry into susceptible cells, orf virus will cause epidermal proliferation which results in increased stratum thickness [27]. Thickness of the stratum will reflect the extent to which the virus replicates and the severity of the tissues involved. The estimation of stratum thickness was more reliable because mean lesion score was quite subjective. Therefore, UPM 2/14 isolate appeared to be more pathogenic than UPM 1/14. Further studies need to be carried out to determine the virulence factors associated with these virus isolates. Like diseases caused by other viruses, there is a need to revisit the probable differences in factors that are influencing pathogenicity, immune responses, and immunity [18, 28]. It would be rather intriguing to determine the capability of orf virus to grow in cell lines of the original host perhaps to affect cell growth in vitro as well. Preparation of appropriate cell lines for virus growth and other possible applications can be easily carried out as described in other studies [29, 30]. Histopathology lesions observed in this study such as keratosis, acanthosis, and ballooning degeneration were consistent with previous report by Kinley et al., [31] for ORFV infection in goats and in rats [15]. Acanthosis was observed in almost all the samples as ORFV encodes viral vascular endothelial growth factor (VEGF), which induces endothelial cell proliferation, vascular permeability, and angiogenesis, thus enhancing epithelial proliferation [32]. However, eosinophilic inclusion bodies were not observed in the skin sections obtained in the present study. According to Barraviera [33], eosinophilic inclusion bodies are demonstrable in the cytoplasm of infected cells but may not be a consistent feature of lesions caused by orf virus.

Dexamethasones are known to suppress the cell-mediated immunity and humoral responses in animals; they act by inhibiting genes that encode for several cytokines, Interleukin 1 (IL-1), IL 2, 3, 4, 5, 6, 8, and TNF-alpha, the most important of which is IL-2. Reduced cytokine production will reduce T cell proliferation [34–36]. They also usually caused B cells to express smaller amounts of IL-2 and IL-2 receptors. This will diminish both B cell clone expansion and antibody synthesis [35]. On the other hand, immunosuppression induced by dexamethasone administration prior to and after ORFV inoculation did not affect the severity of the lesions observed in mice. This contrasts with our recent study in rat model, where dexamethasone-treated rats infected with anyone UPM ORFV isolates showed higher lesion scores and thickness of stratum spinosum than non-dexamethasone-treated rats. The difference between the two studies could be attributed to the difference in response of the mice to the dexamethasone since the duration of dexamethasone administration was short. Prolonged administration of dexamethasone was shown to inhibit T cell proliferation as well as cytokine production from activated CD4+ T cells [37]. CD4+ T cells have been shown to play a critical role in ORFV clearance [38]. As ORFV lesions are normally confined or localized to certain parts of animal bodies, the detail roles of humoral and immune cells for virus clearance and disease recovery should be defined as for other infectious diseases [28, 39].

An approach to employ the two isolates of orf virus in this study (UPM1/14 and UPM2/14) in two different treatment groups was to evaluate possible differences in the pathogenicity of both isolates before subsequent study under the influence of dexamethasone conducted. The use of dexamethasone to induce immunosuppression is not only confined to the study of viruses but also in parasitic diseases such as toxocariasis, where higher larval burdens have been observed [40–42]. This study showed that the administration dexamethasone from days 3 to 14 after inoculation did not alter the course and duration of the development of lesions induced by the orf virus. First, it was found that treatment with dexamethasone did not lessen the clinical signs in orf virus-infected mice, such as altered ruffled fur, weight loss, and laboured respirations. Both dexamethasone-treated and dexamethasone-untreated orf virus-infected BALB/c mice presented the same duration and course of clinical signs with similar severity. Continuous daily treatment with dexamethasone also did not noticeably affect the amount of virus recovered from infected mouse. Therefore, the present data demonstrate that the administration of dexamethasone from days 2 to 7 after orf virus infection does not affect the progression neither effect severity of lesions induced by the virus in mouse model. Xu et al. [16] and Van Woensel et al. [43] have demonstrated that a daily administration of dexamethasone (0.6 mg·kg^−1^·day^−1^) had a beneficial effect in patients with bronchiolitis caused by a respiratory syncytial virus infection. Another recent work has stated and suggested that a lower dose of steroidal compound (1–2 mg·kg^−1^·day^−1^) for a more extended period might benefit the animal while reducing the potential for systemic side-effects [16, 17]. The current results show that daily administration of 5 mg·kg^−1^ dexamethasone on days 3–7 days after infection did not inhibit the development of acute lesions by orf virus infection in mice. Both Malaysian isolates of orf viruses produced similar lesion based on scoring as reported in our recent studies [20]. As such ORFV UPM1/14 isolate was chosen and evaluated for its pathogenicity together with administration of dexamethasone as this mimic's animals under stress situation [16, 26]. In this study, ORFV was detected from the skin samples of experimentally infected mice through PCR amplification targeting at two important genes which were B2L and F1L. This agrees with previous work by Abdullah et al., [6], whom reported the relevance of F1L and B2L genes in the identification and molecular characterization of caprine Orf virus. PCR technique is well known to produce quick confirmatory test for ORFV. This method is recommended for proper detection and identification of pathogens by using specific gene; hence this successful confirmation of positive cases by PCR agrees with [21, 44, 45] whom both in separate studies confirmed CE virus detection by using major genes essential for orf virus replication. An amplified gene product can be used as important genetic materials for further genetic analysis and gene expression studies [46–49] especially for their gene functions. This approach has been adopted for studies on other viruses such as herpes viruses, bacteria, and fungi (Balakrishnan et al., 2017 [50]).

## 5. Conclusion

Intradermal inoculation of Malaysian ORFV isolates in experimental Balb/c mice produced gross and histopathological lesions in mice. Variation in sites of inoculation had no significant effect on pathogenicity of ORFV UPM isolates in mice. Interestingly, dexamethasone administration prior to and after viral inoculation did not have a significant effect on the disease development, progression, and ORFV-associated lesion in mice.

## Figures and Tables

**Table 1:** Scoring protocol for clinical signs.

**Clinical sign**	**Scoring**	**Interpretation**
**Ruffled fur**	0	Normal fur
	2	Ruffled fur at head
	4	Ruffled fur at head and thorax
	6	Ruffled fur at head, thorax and abdomen
**Ocular discharge**	0	Normal eyes
	2	Discharge at upper eyelid
	4	Discharge at upper and lower eyelid
	6	Discharge at upper, lower eyelid and medial canthus
	8	Discharge at upper, lower eyelid, medial and lateral canthus
**Level of alertness**	0	Alert
	1-5	(Number of mice reduced in alertness)
**Clinical Lesion(s)**	2-8	2=hyperemia, 4=macule, 6=papule, 8=vesicle/scab

**Table 2:** Mean lesion scores in different inoculation sites of mice inoculated with ORFV isolates (UPM1/14 and UPM2/14) after 14 days.

**Group**	**Dorsum**	**Ear pinna**	**Labial commissure**
Group 1: UPM1/14	0.048±0.015^a^	0.067±0.020^a^	0.034±0.009^a^
Group 2: UPM2/14	0.052±0.017^a^	0.043±0.015^a^	0.019±0.008^a^
Control Group	0.000±0.000^b^	0.000±0.000^b^	0.000±0.000^b^

Values are expressed as mean±SE

Means in the same column with different superscript are significantly different at p<0.05.

**Table 3:** Total mean lesion scores in mice inoculated with ORFV isolates (UPM1/14 and UPM2/14) after 14 days.

**Group**	**Total mean lesion score**
Group 1: UPM1/14	0.049±0.008^a^
Group 2: UPM2/14	0.038±0.008^a^
Control Group	0.000±0.000^b^

Values are expressed as mean ± SE.

Means in the same column with different superscript are significantly different at p<0.05.

**Table 4:** Mean scores of clinical signs observed in dexamethasone treated and non-treated groups after 6 days of inoculation.

**Clinical Sign**	**Dexamethasone Tx**	**Non-Dexamethasone Tx**	**Control**
Ruffled hair coat	0.311±0.108^a^	0.000±0.000^b^	0.000±0.000^b^
Alertness	0.292±0.136^a^	0.000±0.000^b^	0.000±0.000^b^

Values are expressed as mean ± SE.

Means in the same row with different superscript are significantly different at p<0.05.

**Table 5:** Total mean lesion scores observed in dexamethasone treated and non-treated groups after 6 days of inoculation.

**Group**	**Total mean lesion score**
Dexamethasone Group	0.093±0.031^a^
Non-Dexamethasone Group	0.126±0.013^a^
Control Group	0.000±0.000^b^

Values are expressed as mean ± SE.

Means in the same column with different superscript are significantly different at p<0.05.

**Table 6:** Mean stratum thickness (*µ*m) in mice inoculated with ORFV isolates (UPM1/14 and UPM2/14) after 14 days of inoculation.

**Group**	**Group 1**	**Group 2**	**Control**
Dorsum	24.40±0.35^a^	27.20±2.85^a^	14.63±0.09^c^
Ear pinna	13.50±0.17^a^	25.50±1.53^b^	6.60±0.31^c^
Labial commissure	24.77±1.92^a^	34.30±1.44^b^	11.00±0.83^c^

Values are expressed as mean ± SE.

Means in the same row with different superscript are significantly different at p<0.05.

## Data Availability

The data used to support the findings of this study are available from the corresponding author upon request.
